# 

**Published:** 1884-02

**Authors:** 


CONTENTS.

| | Page. | | Page. |
|---|---|---|---|
| A Simple Remedy | 19 | Checked Perspiration | 32 |
| Health Maxims | 20 | Cases of Longevity | 33 |
| Health is a Duty | 20 | An Object in View | 34 |
| Exercise | 25 | Bacteria in Mountain Air | 35 |
| Health | 27 | Good Food and Good Digestion | 35 |
| Heart Affections | 28 | Mothers | 36 |
| House Building | 29 | Milk and Contagion | 36 |
| Catarrh | 30 | Typhoid Fever in New York | 38 |
| Wakefulness | 31 | | |

